# The Association between the Diversity of Coenzyme Q10 Intake from Dietary Sources and the Risk of New-Onset Hypertension: A Nationwide Cohort Study

**DOI:** 10.3390/nu16071017

**Published:** 2024-03-31

**Authors:** Suming Dai, Zezhong Tian, Dan Zhao, Ying Liang, Zepei Zhong, Yixuan Xu, Shanshan Hou, Yan Yang

**Affiliations:** 1 School of Public Health (Shenzhen), Shenzhen Campus of Sun Yat-sen University, Shenzhen 518107, China; daism7@mail2.sysu.edu.cn (S.D.); tianzzh3@mail.sysu.edu.cn (Z.T.); zhaod39@mail2.sysu.edu.cn (D.Z.); zhongzp@mail2.sysu.edu.cn (Z.Z.); xuyx63@mail2.sysu.edu.cn (Y.X.); houshsh3@mail2.sysu.edu.cn (S.H.); 2 Guangdong Engineering Technology Center of Nutrition Transformation, Sun Yat-sen University, Shenzhen 518107, China; 3 Guangdong Provincial Key Laboratory of Food, Nutrition and Health, Sun Yat-sen University, Guangzhou 510080, China; 4 The Eighth Affiliated Hospital of Sun Yat-sen University, Shenzhen 518033, China; liangy228@mail2.sysu.edu.cn

**Keywords:** food diversity, dietary coenzyme Q10, hypertension

## Abstract

Coenzyme Q10 (CoQ10) is a food active component with blood-pressure-improving properties. However, the association between the variety and quantity of different sources of dietary CoQ10 and new-onset hypertension remains uncertain. We aimed to investigate the associations between the diversity and quantity of CoQ10 intake from eight major food sources and new-onset hypertension risk. A total of 11,489 participants were included. Dietary intake was evaluated via three consecutive 24 h recalls and household food inventory. The diversity score of CoQ10 sources was calculated by the sum of food groups consumed in the ideal range. Cox proportional hazard models were used for evaluating their associations with hypertension. Model performance was assessed by ROC analyses and 200-times ten-fold cross-validation. The relationships between CoQ10 and hypertension were U-shaped for meat, egg, vegetable, and fruit sources, inverse J-shaped for fish, and nut sources, and L-shaped for dairy products sources (all *p*-values < 0.001). A higher diversity score was associated with lower hypertension risk (HR (95% CI): 0.66 (0.64, 0.69)). The mean areas under the ROC curves for 6, 12 and 18 years were 0.81, 0.80 and 0.78, respectively. There is a negative correlation between the diversity of CoQ10 with moderate intake from different sources and new-onset hypertension. One diversity score based on CoQ10 was developed.

## 1. Introduction

The global prevalence of hypertension remains remarkable, affecting around 1.3 billion adults in 2019 [1]. High systolic blood pressure is the leading risk factor for attributable premature cardiovascular deaths worldwide [2]. The significant increase in the prevalence of and the contribution of hypertension to cardiovascular deaths was largely displayed in developing countries [1,2]. In China, around 2.67 million cardiovascular deaths were attributed to hypertension in 2018 [3].

Among the major risk factors for hypertension, unhealthy diets are undeniably among the most important ones [4]. The management of hypertension could be achieved by certain changes in the composition of diets, including the consumption of specific food groups and intake of food active components [5,6]. Coenzyme Q10 (CoQ10) is one representative food active component with a lot of benefits for cardiovascular health [7,8,9]. Randomized controlled trials and meta-analysis of them endorsed solid conclusions that CoQ10 supplementation has beneficial effects on the control of blood pressure in patients with cardio-metabolic diseases [10]. Most CoQ10 supplements are expensive and difficult to promote in the daily lives of the entire population. The exogenous acquisition methods of CoQ10 include various natural foods in addition to nutritional supplements [11,12]. Previous studies have also found a significant non-linear correlation between the total intake of CoQ10 from dietary sources and risk factors related to cardiovascular disease, such as *C*-reactive protein [13]. However, it is unclear whether and how dietary CoQ10 sources are related to the primary prevention of hypertension.

In addition, there are significant differences in the content of CoQ10 in various foods [12]. Previous studies have found a significant negative correlation between the diversity of dietary intake sources for specific nutritional components and the risk of hypertension (such as fiber and protein) [14,15]. However, there is currently no study exploring the association and potential dose–response relationship between CoQ10 intake from different types of food sources and new-onset hypertension. There is also no study exploring the relationship between the diversity of CoQ10 intake from different dietary sources and new-onset hypertension, which would be helpful in order to more accurately guide individuals to use dietary CoQ10 to help prevent the new-onset hypertension.

Accordingly, we aimed to investigate the relationship between the diversity and quantity of CoQ10 intake from eight main food sources (including meats, eggs, dairy products, fishes, plant oils, nuts, vegetables and fruits) and new-onset hypertension, and to establish one new diversity score of dietary CoQ10 sources via a large prospective cohort.

## 2. Materials and Methods

### 2.1. Study Participants

The participants for this study were from China Health and Nutrition Survey (CHNS). The CHNS was an ongoing open cohort conducted by an international team of researchers with diverse academic backgrounds. It was started in 1989 and followed up every 2 to 4 years, using a multistage random clustering sampling. More details of the design are available in prior studies [16]. The original data and study materials can be found on the CHNS official website (https://www.cpc.unc.edu/projects/china (accessed on 5 September 2021)). This survey was approved by the institutional review boards of the University of North Carolina at Chapel Hill and the National Institute for Nutrition and Health and the Chinese Center for Disease Control and Prevention. Written informed consent was obtained from each participant ahead of survey.

**Figure 1:**
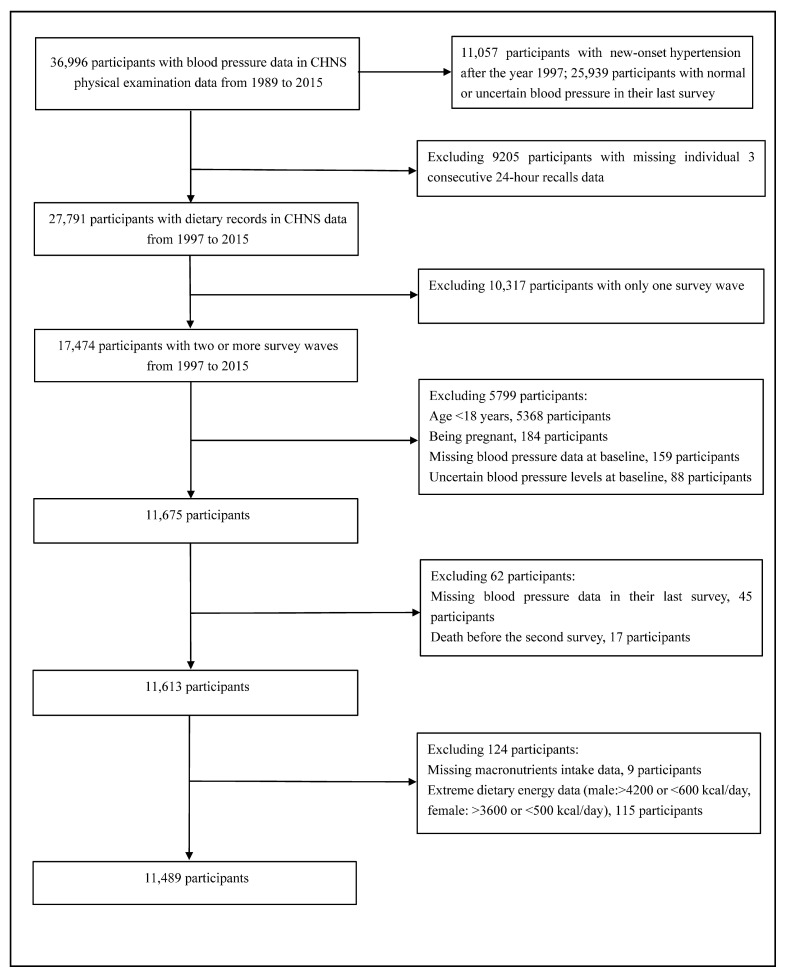
The flow chart of study participants.

The current study design was a prospective cohort based on seven-round surveys of CHNS from 1997 to 2015. Inclusion was given to participants meeting all of the following criteria: (1) taking at least two rounds of the CHNS; (2) aged no less than 18 years old; (3) with dietary records and reasonable total energy intake; and (4) with blood pressure data. Participants were excluded if they met one or more of the following characteristics: (1) younger than 18 years or pregnant; (2) missing dietary or blood pressure data; (3) suffering from hypertension at baseline; or (4) with implausible energy intake (male: <600 or >4200 kcal/day; female: <500 or >3600 kcal/day). The flow chart is shown in Figure 1.

### 2.2. Dietary Assessment

In the CHNS, dietary data were obtained from three consecutive 24 h recalls at individual levels combined with 3-day food-weighed records at household levels. The reliability of such methods to assess energy and nutrient intake was validated in CHNS [17]. Daily nutrient intake and total energy were calculated by summing specific contributions across all food items using the China food composition tables (CFCT) in each round of the survey. In our analyses, the cumulative average intake of each nutrient, total energy, CoQ10 and food groups was calculated from baseline to the end point for each participant in order to better assess the long-term dietary intake status.

### 2.3. Calculation of Diversity Score of CoQ10 Sources

Food items containing CoQ10 were distinguished using existing knowledge from the published literature [12,18,19]. Then, they were categorized into 8 major groups based on the consideration of nutrient, culinary similarities and CoQ10 contents. The eight food groups were within larger categories of animal foods (including meats and their processed foods, fishes and shellfish, eggs, dairy products) and plant foods (including plant cooking oils, nuts and seeds, vegetables and their processed foods, fruits and their processed foods). Detailed food items in each food group were listed in Supplementary Table S1. In each food group, the daily intake was calculated by adding up the intake of all food items within that group.

We created a food diversity score using a procedure similar to the one used by Mengyi Liu et al. [20]. Non-consumers were assigned a score of 0. The consumers were divided into quartiles by intake of each food group. When the proportion of non-consumers was large, the consumers would be divided into tertiles. Each quartile/tertile was assigned a score of 1 or 0 on the basis of its association with new-onset hypertension risk. The observed range of this score was 0 to 8.

### 2.4. Ascertainment of Follow-Up Events

The follow-up time of participants free of hypertension was calculated as the years between entry to the study and the date of occurrence of censor (lost to follow-up, death, or end of follow-up)—whichever occurred first. For participants first identified with hypertension, the person year was the duration between baseline and the midpoint of this and the last survey.

In the study, hypertension cases were identified via blood pressure measurement (the average systolic blood pressure (SBP) ≥ 140 mmHg or the average diastolic blood pressure (DBP) ≥ 90 mmHg) or self-reported use of anti-hypertension medicine or the physician diagnosis of hypertension [21].

### 2.5. Basic Information Collection

Socio-demographic characteristics and lifestyle factors were collected through qualified questionnaires. Waist circumference, body weight and height were measured following stringent anthropometric procedures. Body mass index (BMI) was calculated by dividing weight (kg) by height squared (m^2^). Physical activity was assessed using metabolic equivalent values multiplied by self-reported time spent in each activity [22]. To minimize within-person variation, the cumulative average values of BMI and physical activity level were calculated and used in our analysis.

### 2.6. Statistical Analyses

The study participants’ characteristics were presented as mean with standard deviation (SD) for continuous variables, and as percentages for categorical variables. The baseline characteristics of participants were compared by dietary total CoQ10 intake quintiles using χ^2^ test for categorical variables and ANOVA or the Kruskal–Wallis test for continuous variables.

The Cox proportional hazard regression model was used to assess the relationship of different CoQ10 food sources and diversity score with the risk of new-onset hypertension. The hazard ratios (HRs) and their corresponding 95% confidence intervals (CIs) were estimated with the lowest quantile serving as the reference group. The quantiles’ median value was used as a quasi-continuous variable in the model for calculating *p* values for trends. Before conducting the above analyses, the proportional hazard assumption was checked using the cox.zph function in R (version 4.1.2). To control for potential confounding factors, the following covariates were included in the multivariable models with the stepwise method—age, sex, BMI, job, education level, north or south region, smoking status, drinking status, baseline SBP, abdominal obesity, physical activity, total energy intake (kcal/day), and intake of other dietary CoQ10 sources. Restricted cubic splines were also employed to explore the potential nonlinear relationship between different CoQ10 food sources and the risk of new-onset hypertension.

Stratified analyses were further performed by prespecified variables to evaluate the potential modifiers of the association between the diversity score of CoQ10 source and new-onset hypertension. Likelihood ratio tests comparing models with and without the interaction term were used to evaluate the interaction.

Several sensitivity analyses were conducted to test the robustness of our primary findings. First, dietary intake adjusted for total energy using the residual method was employed in the models [23,24]. Second, the follow-up time of participants identified with hypertension was recalculated as the years from the baseline to the first hypertension diagnosis. Third, participants who were identified with hypertension in the first two years of follow-up were excluded. Fourth, the analyses were conducted among all participants after multiple imputations of missing covariates by chained equation were performed. Fifth, we reassessed the association between the diversity score of CoQ10 source and new-onset hypertension risk after the removal of any one kind of dietary CoQ10 sources.

A receiver operating characteristic analysis was conducted to help evaluate the model performance at different time points. The area under the receiver operating characteristic curve (AUC) was calculated. The concordance of the predicted risk and the observed frequency of hypertension was visualized. The results were under ten-fold cross-validation for 200 times.

All statistical analyses were performed by R (version 4.1.2, http://www.R-project.org/ (accessed on 14 November 2021)). A two-tailed *p* value < 0.05 was considered statistically significant.

## 3. Results

### 3.1. Study Participants and Basic Characteristics

In the current study, 11,489 participants meeting the criteria were included. These participants were followed-up for a total of 94,557 person years, with a median follow-up period of 6.2 years. A total of 3904 participants were identified with new-onset hypertension during this period.

The characteristics of the study participants are presented in Supplementary Table S2 according to total CoQ10 intake quintiles. The mean (SD) age of them was 41.0 (13.8) years. Most of them were females (54.3%). The median total CoQ10 intake was 4.3 (IQR: 2.8–6.4) mg/d (Supplementary Table S3). With a higher intake of total CoQ10, the participants were more likely to be young, males and alcohol drinkers, and have higher BMI, abdominal obesity, higher education levels, and higher intake of fat and protein, while they were less likely to be farmers and illiterate.

### 3.2. Association between Different Food Sources of CoQ10 and New-Onset Hypertension Risk

**Figure 2:**
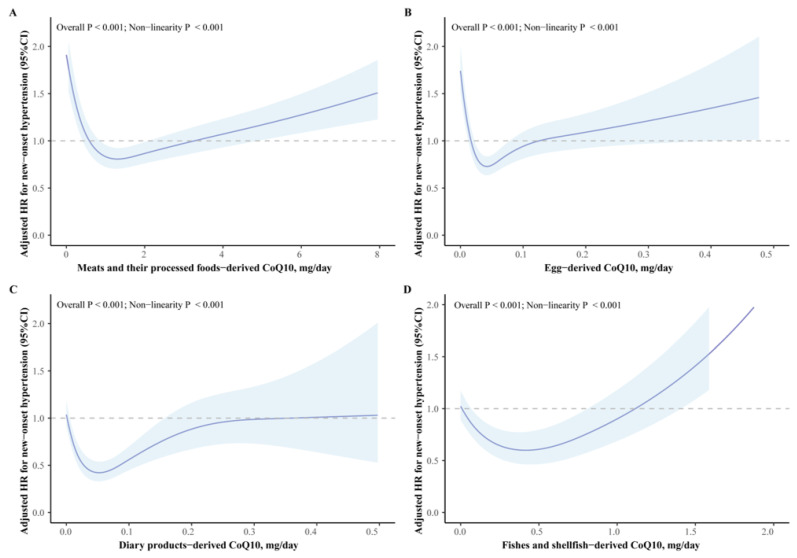
The relations of animal-food-derived CoQ10 and new-onset hypertension. Adjusted for age, sex, BMI, job, education level, region, smoking status, alcohol drinking status, baseline SBP, physical activity, abdominal obesity, total energy intake (kcal/day), and mutual adjustments for CoQ10 from other sources (mg/day). The dark blue curve represents the estimated value, and the light blue area represents 95% CIs. The gray dashed line represents the reference line with HR = 1. (**A**) meat- and their processed-food-derived CoQ10; (**B**) egg-derived CoQ10; (**C**) dairy-product-derived CoQ10; (**D**) fish- and shellfish-derived CoQ10.

**Figure 3:**
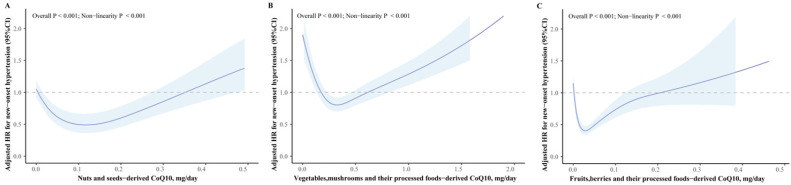
The relations of plant-food-derived CoQ10 and new-onset hypertension. Adjusted for age, sex, BMI, job, education level, region, smoking status, alcohol drinking status, baseline SBP, physical activity, abdominal obesity, total energy intake (kcal/day), and mutual adjustments for CoQ10 from other sources (mg/day). The dark blue curve represents the estimated value, and the light blue area represents 95% CIs. The gray dashed line represents the reference line with HR = 1. (**A**) nut- and seed-derived CoQ10; (**B**) vegetable- and their processed-food-derived CoQ10; (**C**) fruit- and their processed-food-derived CoQ10.

Among the eight dietary CoQ10 sources, there is a U-shaped nonlinear dose–response relationship between the CoQ10 intake from meat, egg, and vegetable sources, and fruit sources and the risk of new-onset hypertension (all nonlinear *p*-values < 0.001, Figure 2A,B and Figure 3B,C). The intake of CoQ10 from fishes and nuts is inversely J-shaped correlated with the risk of new-onset hypertension, while the intake of CoQ10 from dairy products is L-shaped correlated with the risk of new-onset hypertension (all nonlinear *p*-values < 0.001, Figure 2C,D and Figure 3A).

**Figure 4:**
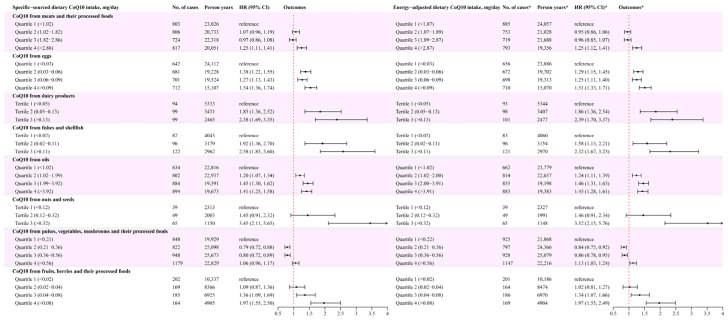
The forest plot of different food sources of CoQ10 and hypertension risk. The intake of consumers was categorized into quartiles or tertiles. The HRs and 95% CIs were shown as the black solid dots and black horizontal solid lines. The red vertical dashed lines represent the reference lines with HR = 1. On the right part of this figure, the intake was adjusted by total energy using the residual method. The symbol of * represents that the results were obtained based on energy-adjusted CoQ10 intake.

After assessing the CoQ10 intake from different sources in quantiles among consumers, the neutral intake of vegetables and their processed foods was associated with a decreased new-onset hypertension risk. In detail, the multivariable adjusted HRs (95% CIs) of hypertension for consumers in the second and third quartile (vs. the lowest) were 0.79 (0.72 to 0.88) and 0.80 (0.72 to 0.89) for a CoQ10 intake source of vegetables and their processed foods. In addition, among consumers, increased intake of other CoQ10 food sources was associated with s higher risk of new-onset hypertension. Fully adjusted HRs (95% CIs) of hypertension for consumers in the highest (vs. the lowest) quantile group of these CoQ10 intake sources were 1.25 (1.11 to 1.41) for meats and their processed foods, 1.54 (1.36 to 1.74) for fishes and shellfish, 2.38 (1.69 to 3.35) for eggs, 2.58 (1.85 to 3.60) for dairy products, 1.41 (1.25 to 1.58) for plant-sourced cooking oils, 3.45 (2.11 to 5.65) for nuts and seeds, and 1.97 (1.55 to 2.50) for fruits and their processed foods (Figure 4).

Accordingly, the ideal intake of eight CoQ10 food groups was as follows: 140–581 g/week for meats and their processed foods, 63–112 g/week for eggs, 280–511 g/week for dairy products, 49–77 g/week for fishes and shellfish, 20–25 g/day for plant oils, 5–18 g/day for nuts and seeds, 171–533 g/day for vegetables and their processed foods, and 105–175 g/week for fruits and their processed foods (Figure 4 and Supplementary Table S1). Similar results were observed when the consumption was adjusted by total energy intake (Figure 4).

### 3.3. Relationship of Diversity Score of CoQ10 Sources and New-Onset Hypertension

Table 1 presents the relationship between the diversity score of CoQ10 sources and new-onset hypertension risk based on a continuous one score increment and the divided four groups. A significant inverse association was observed between them in model 1 and model 2. That is, with a one score increment, the HR (95% CIs) of new-onset hypertension was 0.65 (0.63 to 0.67) in model 1 and 0.66 (0.64 to 0.69) in model 2. When the score was categorized into different groups, the HRs (95% CIs) in model 2 were 0.51 (0.47 to 0.55), 0.43 (0.38 to 0.49) and 0.28 (0.21 to 0.39) for participants with a diversity score of 2, 3 and ≥4, compared to those with a score <2.

The results were not materially altered when the dietary intake was total-energy adjusted, the follow-up time for participants identified with new-onset hypertension was from the baseline to the first diagnosis, or after multiple imputations of missing covariates, including the removal of any one kind of dietary CoQ10 sources from the total score (Supplementary Tables S4–S6). The results of stratified analyses are shown in Table 2. The diversity scores of CoQ10 sources and new-onset hypertension were significantly inversely correlated in all pre-specified subgroups. The *p* values for interaction were <0.05 for sex, drinking status and total energy intake. But considering the similar trends in both subgroups, the implications warranted further investigation.

**Figure 5:**
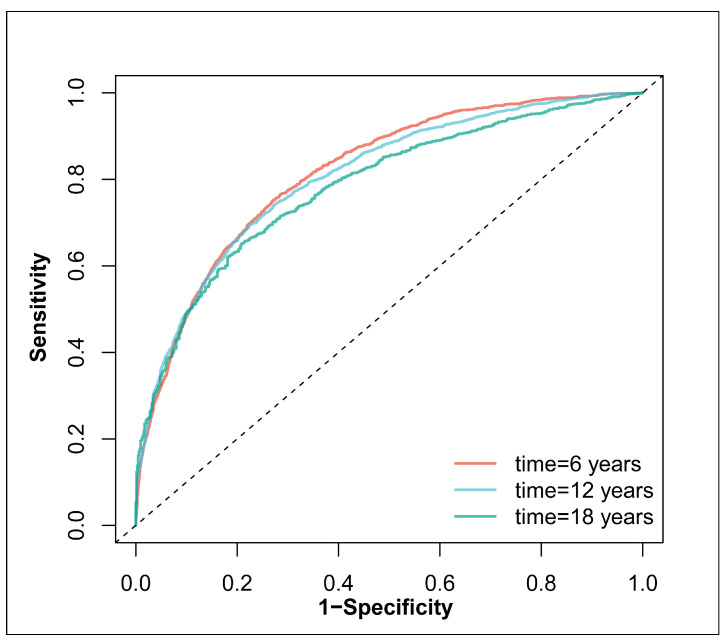
The ROC curves at different time points. The ROC curves are illustrated in red, blue and green for the time six years, twelve years and eighteen years, based on model 2 with a diversity score of CoQ10 source as one continuous variable. The dashed line represents the reference line, the area under which is 0.5. Abbreviations: ROC = receiver operating characteristic curve; AUC = area under the curve.

**Figure 6:**
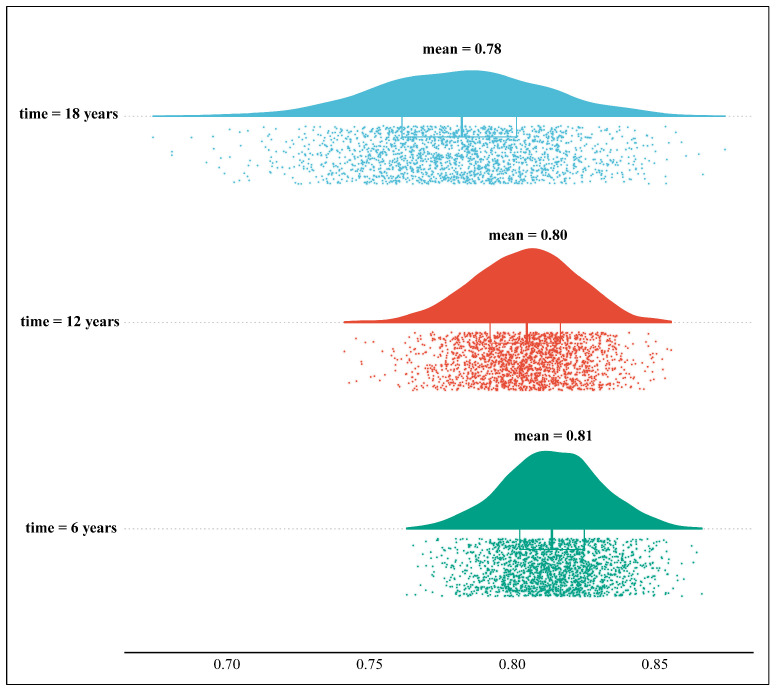
The visualization of 200-times 10-fold cross-validation of this cohort. At the time points of six years, twelve years and eighteen years, the cohort was internally validated via the method of ten-fold cross validation for 200 times. The distribution of 2000 values of AUC at each time point is shown in this figure.

The AUCs for model 2 with scores as continuous variables were 0.82 (0.81 to 0.83), 0.81 (0.80, 0.82) and 0.78 (0.77 to 0.80) at 6 years, 12 years and 18 years, respectively (Figure 5). The calibration curve between the predicted risk and observed frequency of new-onset hypertension is shown in Supplementary Figure S1. After 10-fold cross-validation for 200 times, the AUCs were not materially altered. The mean AUCs for 6, 12 and 18 years were 0.81, 0.80 and 0.78, respectively (Figure 6).

## 4. Discussion

### 4.1. Principal Findings

Our study found that there were differences in the association between CoQ10 from different food sources and new-onset hypertension. Further analysis indicated that there was a negative correlation between the diversity of CoQ10 with moderate intake from different dietary sources and new-onset hypertension. Considering the complexity of diet, our current study developed a simple and practical diet score to assess the diversity of CoQ10 sources and its association with new-onset hypertension in Chinese adults. Differently, this novel score originated from CoQ10 with blood-pressure-lowering properties and its existence in a variety of natural foods. We found that the diversified dietary pattern based on a moderate intake of dietary CoQ10 sources was significantly associated with a decreased new-onset hypertension risk. Shown in the scores for simplicity, a per score increment in diversity score of CoQ10 sources was associated with a 34% lower risk of new-onset hypertension.

The ideal intake levels of different CoQ10 food sources were reassessed, thus providing new evidence for the association between some food groups and new-onset hypertension risk to some extent. For vegetables, our finding was in line with one recent meta-analysis of prospective studies suggesting an inverse association between vegetable intake and hypertension risk in Asian people [25]. However, one meta-analysis of prospective studies published after this study found that the reduced risk of hypertension for high versus low vegetable intake was insignificant [26]. This discrepancy could be attributed to different study regions and mixed vegetable subtypes. For dairy products, our analyses showed that higher consumption of dairy products containing CoQ10 seemed to have a detrimental effect on preventing hypertension. One reason may be that some specific types of dairy products are technologically processed or have higher fat contents, which were previously reported to have a positive association with hypertension. As in the case of butter, cross-sectional analysis indicated that DBP was higher across increasing quartiles of intake [27]. In addition, a positive association between processed cheese consumption and risk of hypertension was observed in a prospective cohort of French women [28]. As for the nonlinear association, the latest meta-analysis of prospective cohort studies also observed a steep, followed by a smooth, L-shaped curve between total dairy product consumption and risk of hypertension [29]. For fish, the recent meta-analysis of prospective studies indicated that the hypertension risk was slightly increased with increasing the fish intake ≤100 g/d and lowered with a fish intake above this value in a nonlinear dose–response analysis [25]. However, no association was observed for each 100 g/d increase in fish intake or high- versus low-intake categories [25]. Additionally, one prospective cohort study conducted after this meta-analysis also found that fish consumption was not associated with hypertension risk [30]. The intake of fish was relatively low in our study participants. Although an increased hypertension risk was found when comparing high versus low fish intake categories of consumers, further studies are required to confirm or refute this finding. For egg, one recent prospective study with a considerable number of participants observed an increased risk of hypertension associated with high egg intake, consistent with our results [31]. Of note, the risk increased linearly up to about seven eggs every week, and then decreased. It was implied that the positive association may be driven by cholesterol [32]. However, the meta-analysis of randomized controlled trials stated that a general conclusion could not be drawn about the effect of egg consumption on blood pressure due to various limitations in relevant studies [33]. Further studies are still needed to provide more evidence.

In addition, accumulating evidence indicated a positive association of red meat and processed meat with hypertension risk [34]. Our study yielded a similar positive relationship, although the contained meat items were rich in CoQ10. However, for nuts and fruits, the results appeared to be in opposition to protective effects on hypertension reported in large prospective cohort studies or meta-analyses of prospective studies [25,26,35]. The discrepancy could be attributed to the probably high fat contents connected with fruits relatively rich in CoQ10 and the limited consumption of included nuts and seeds among our participants. This result could imply the importance of considering the quality of foods consumed, even if these are plant foods.

Apart from the aforementioned food groups, there were some differences in food groups we focused on compared to other diet scores. To the best of our knowledge, there has not been any prospective studies conducted to explore the association of the food group oils with new-onset hypertension risk. Single-sourced oil, such as olive oil, sesame oil and fish oil, was investigated by randomized controlled trials on the efficacy of lowering the blood pressure among different populations [36,37,38]. For the general population, one Mediterranean prospective cohort study denoted that olive oil was associated with a reduced risk of hypertension only among male participants [39]. But the sample size and the follow-up time were limited, along with the low cumulative incidence of hypertension. Our results implied that plant-sourced oils exerted a detrimental effect on hypertension incidence. In light of the limited evidence, further research is warranted to clarify this relation.

The manner of constructing an a priori dietary diversity score was demonstrated to be successful for the study in [40]. Similar to some previous diet scores, such as the Mediterranean Diet Score, Dietary Approaches to Stop Hypertension (DASH) Score, and Planetary Health Diet Score, our score scheme used an unweighted score method [41]. At the same time, the food groups contained in this current score were mostly like other diet scores and mostly covered food types consumed in daily meals [42,43,44]. However, given the controversial results of the association of some food groups and new-onset hypertension risk, each food group was not scored based on quintile ranking (1 to 5 score corresponding to a range from the lowest quintile to the highest quintile intake for prespecified healthy foods, and the reverse for unhealthy foods). We combined the quantity and variety, which meant every 1 score represented eating the ideal amount from one food group. The score became higher when more food groups were consumed at the ideal level.

Furthermore, the present diversity score of CoQ10 sources appeared to be well applicable in Chinese adults. Specifically, the association between this score and the risk of new-onset hypertension was significant, and the AUC levels were satisfactory in the initial analysis and subsequent internal validation. Some other priori diet scores were also associated with decreased hypertension risk, but the extent was limited. For instance, the pooled analysis of prospective studies indicated that high adherence to DASH was only associated with a 19% lower risk of hypertension compared to low adherence [45]. Among the included studies, one was conducted in Chinese adults. The results showed that subjects with the highest quintile of DASH score had a 15% lower risk of incident hypertension compared to the lowest quintile group, which implied that the Chinese population may not benefit from the DASH diet unless they strongly adhere to it [46]. Additionally, the association of the Mediterranean diet score and risk of hypertension was also previously investigated among Chinese general adults [47]. In that prospective study, the efficacy of preventing new-onset hypertension was similar to our score, but was only limited to the group with a constantly high or increasing Mediterranean diet adherence. Thus, it can also be seen that adherence to a healthy diet for the primary prevention of hypertension is hard to fulfill.

### 4.2. Strengths and Limitations

Our findings provided an alternative way to achieve the dietary goal of preventing hypertension. In our study, one score increment was related to a significant reduction in new-onset hypertension risk. That is, gradually moving towards a healthy diet by progressively controlling the intake of more food groups within the ideal range is also helpful to prevent hypertension. Compared to highly adhering to one healthy diet from the beginning or restricting one’s intake to only so-called absolutely healthy foods, this point could be more conducive to motivating the public to change their habits and gradually move towards a balanced diet. Accordingly, our study suggested incremental dietary changes and supported that a diet of diversity and moderation is also an ideal or even better diet for hypertension prevention. In addition, this prospective epidemiological study could provide a simple way to evaluate one person’s diet and its propensity towards a risk of new-onset hypertension. Specific food items in each food group were simplified according to CoQ10 contents. And the use of separate cut-offs for each food group in our scoring scheme provided a distinctive and meaningful indicator of food amounts associated with hypertension prevention.

Nevertheless, some potential limitations need to be considered in the current study. First, the diet was assessed by self-reported 3-day 24 h recall questionnaires, as in many large cohort studies. Although their reliability was validated, some sources of error may still remain. Second, the residual confounding in observation studies, usually arising from unknown or imprecisely measured covariates, cannot be completely avoided. The results should be still considered with caution. Third, whether the score can be applicable on a larger scale is still undetermined. However, we applied sensitivity analysis and internal validation methods to indicate that the results were robust.

## 5. Conclusions

In conclusion, our study indicated that there was a negative correlation between the diversity of CoQ10 with moderate intake from different dietary sources (including meats, eggs, dairy products, fishes, plant oils, nuts, vegetables and fruits) and the risk of new-onset hypertension in Chinese adults. Consuming moderate foods from various dietary CoQ10 sources could help to prevent the occurrence of hypertension. In addition, we established a simple diversity score of dietary CoQ10 intake to guide individuals in reducing the risk of hypertension.

## Figures and Tables

**Table 1:** The association between food diversity score based on dietary CoQ10 and new-onset hypertension.

Diversity Score	Model 1 ^a^		Model 2 ^b^	
	HR (95% CI)	*p* Value	HR (95% CI)	*p* Value
As continuous variable				
	0.65 (0.63, 0.67)	<0.001	0.66 (0.64, 0.69)	<0.001
As categorical variable				
Group 1 (<2)	Ref		Ref	
Group 2 (2)	0.49 (0.46, 0.54)	<0.001	0.51 (0.47, 0.55)	<0.001
Group 3 (3)	0.41 (0.36, 0.46)	<0.001	0.43 (0.38, 0.49)	<0.001
Group 4 (≥4)	0.29 (0.22, 0.37)	<0.001	0.28 (0.21, 0.39)	<0.001
*p* for trend	<0.001		<0.001	

^a^ Model 1: adjusted for age, sex and BMI. ^b^ Model 2: adjusted for age, sex, BMI, job, education level, region, smoking status, alcohol drinking status, baseline SBP, physical activity, abdominal obesity, total energy intake (kcal/day), total dietary CoQ10 intake (mg/day).

**Table 2:** Stratified analyses of the association between diversity score and new-onset hypertension.

	Adjusted Model		
Subgroups	HR (95% CI)	*p* Value	*p* for Interaction
**Age, years**			0.625
<60	0.66 (0.64, 0.69)	<0.001	
≥60	0.63 (0.58, 0.70)	<0.001	
**Sex**			0.043
Male	0.64 (0.61, 0.68)	<0.001	
Female	0.68 (0.65, 0.72)	<0.001	
**BMI, kg/m^2^**			0.557
<24	0.67 (0.63, 0.70)	<0.001	
≥24	0.66 (0.62, 0.69)	<0.001	
**Physical activity, Mets⸱h/wk**			0.102
Low	0.69 (0.64, 0.73)	<0.001	
Moderate	0.68 (0.64, 0.72)	<0.001	
High	0.61 (0.57, 0.65)	<0.001	
**Smoking status**			0.173
No	0.67 (0.64, 0.70)	<0.001	
Yes	0.64 (0.60, 0.68)	<0.001	
**Drinking status**			0.014
No	0.68 (0.65, 0.71)	<0.001	
Yes	0.63 (0.60, 0.67)	<0.001	
**Baseline SBP stages**			0.403
<120	0.66 (0.63, 0.70)	<0.001	
≥120	0.66 (0.62, 0.70)	<0.001	
**Abdominal obesity**			0.766
No	0.66 (0.64, 0.69)	<0.001	
Yes	0.66 (0.62, 0.71)	<0.001	
Unknown	0.70 (0.52, 0.93)	0.014	
**Total energy intake, kcal**			0.013
<2110 (median)	0.68 (0.64, 0.71)	<0.001	
≥2110	0.63 (0.60, 0.66)	<0.001	

If not stratified, adjusted for age, sex, BMI, job, education level, region, smoking status, alcohol drinking status, baseline SBP, physical activity, abdominal obesity, total energy intake (kcal/day), total dietary CoQ10 intake (mg/day).

## Data Availability

The original datasets are available on the CHNS official website (https://www.cpc.unc.edu/projects/china (accessed on 5 September 2021)). The datasets used and analyzed in the current study are available from the corresponding author on reasonable request.

## Supplementary Materials

The following supporting information can be downloaded at: https://www.mdpi.com/article/10.3390/nu16071017/s1, Table S1: FDSC10 components and scoring standards. Table S2. The characteristics of study participants by total CoQ10 intake quintiles. Table S3. Dietary intake of different-sourced CoQ10 and energy-adjusted CoQ10 (mg/d) in study participants. Table S4. The association between diversity score of dietary CoQ10 source and new-onset hypertension. Table S5. Sensitivity analyses for the association between diversity score of dietary CoQ10 source and new-onset hypertension. Table S6. The association between diversity score of CoQ10 and new-onset hypertension after the removal of any one kind of dietary CoQ10 sources. Figure S1. The calibration curves at different times.
