# Supplementary material for: The Association between the Diversity of Coenzyme Q10 Intake from Dietary Sources and the Risk of New-Onset Hypertension: A Nationwide Cohort Study
Source: Nutrients. 2024 Mar 31;16(7):1017. doi: 10.3390/nu16071017 (PMC11013836; doi:10.3390/nu16071017)
Supplement: Supplementary file 1 [file nutrients-16-01017-s001.zip › Supplementary File.pdf]

Supplementary Materials

Supplementary Table S1. FDSC10 components and scoring standards.

Supplementary Table S2. The characteristics of study participants by total CoQ10 intake quintiles.

Supplementary Table S3. Dietary intake of different-sourced CoQ10 and energy-adjusted CoQ10 (mg/d) in study participants.

Supplementary Table S4. The association between diversity score of dietary CoQ10 source and new-onset hypertension.

[a] The CoQ10 intake was adjusted by total energy using the residual method.

[b] Model 1: adjusted for age, sex and BMI.

[c] Model 2: adjusted for age, sex, BMI, job, education level, region, smoking status, alcohol drinking status, baseline SBP, physical activity, abdominal obesity, as well as total energy intake (kcal/day), total dietary CoQ10 intake (mg/day).

Supplementary Table S5. Sensitivity analyses for the association between diversity score of dietary CoQ10 source and new-onset hypertension.

[a] Model 2: adjusted for age, sex, BMI, job, education level, region, smoking status, alcohol drinking status, baseline SBP, physical activity, abdominal obesity, as well as total energy intake (kcal/day), total dietary CoQ10 intake (mg/day).

[b] Analysis 1: The follow-up time of participants with new-onset hypertension were recalculated as the years from the baseline to the first hypertension diagnosis.

[c] Analysis 2: Participants who developed hypertension in the first two years of follow-up were excluded.

[d] Analysis 3: The analyses were conducted among all participants after multiple imputations of missing covariates by chained equation was performed

Supplementary Table S6. The association between diversity score of CoQ10 and new-onset hypertension after the removal of any one kind of dietary CoQ10 sources.

[a] Model 1: adjusted for age, sex and BMI.

[b] Model 2: adjusted for age, sex, BMI, job, education level, region, smoking status, alcohol drinking status, baseline SBP, physical activity, abdominal obesity, as well as total energy intake (kcal/day), total dietary CoQ10 intake (mg/day).

Supplementary Figure S1. The calibration curves at different times.

Supplementary Table S1. FDSC10 components and scoring standards.

Type of food	Components	Intake range for score
Meats and their processed foods	Beef heart, beef liver, beef shoulder, beef thigh, beef tenderloin; pork heart, pork liver, pork sirloin, pork lard; chicken heart, chicken liver, chicken high, chicken cheat, chicken wing	140-581g, weekly
Eggs	Chicken egg, chicken yolk	63-112g, weekly
Dairy products	Butter, cheese Emmental, cow milk (3.6% fat), yogurt, cream (35% fat), curd (35% fat)	280-511g, weekly
Fishes and shellfish	Horse mackerel, sardine, herring, tuna canned, rainbow trout, eel, salmon, scallop, etc.	49-77g, weekly
Plant-sourced cooking oils	Soybean oil, corn oil, olive oil, rapeseed oil, peanut oil, sesame oil, cottonseed oil, sunflower oil, safflower oil	20-25g, daily
Nuts and seeds	Peanuts, sesame seeds, pistachio nuts, walnuts, hazelnuts, almond, chestnuts	5-18g, daily
Vegetables and their processed foods	Soybean green, soybean whole, broccoli, rape, spinach, cauliflower, sorrel, Chinese cabbage, sweet potato, sweet pepper, etc.	171-533g, daily
Fruits and their processed foods	Avocado, blackcurrant, strawberry, orange, grapefruit, apple, banana, persimmon, kiwi	105-175g, weekly

Supplementary Table S2. The characteristics of study participants by total CoQ10 intake quintiles.

Characteristics	Quintile					*p* value
	Q1 (<2.5 mg/d)	Q2 (2.5-3.7 mg/d)	Q3 (3.7-5.0 mg/d)	Q4 (5.0–7.0 mg/d)	Q5 (>7.0 mg/d)	
N	2298	2298	2298	2298	2297	
**Age, years**	42.2 ±14.8	41.1 ±13.8	40.7 ±13.4	40.3 ±13.3	40.3 ±13.4	<0.001
**Male, n (%)**	955 (41.6)	992 (43.2)	1042 (45.3)	1132 (49.3)	1132 (49.3)	<0.001
**Systolic blood pressure, mmHg**	113.6 ±11.7	113.6 ±12.0	113.3 ±11.6	114.2 ±11.2	114.8 ±10.7	<0.001
**Diastolic blood pressure, mmHg**	73.4 ±8.1	73.8 ±8.1	73.6 ±7.7	74.5 ±7.6	75.3 ±7.5	<0.001
**Body mass index, kg/m^2**	22.8 ±3.3	22.7 ±3.3	22.7 ±3.1	23.0 ±3.1	23.3 ±3.2	<0.001
**Abdominal obesity, n (%)**	566 (24.6%)	574 (25.0%)	510 (22.2%)	551 (24.0%)	613 (26.7%)	<0.001
**North region, n (%)**	910 (39.6)	796 (34.6)	675 (29.4)	908 (39.5)	1279 (55.7)	<0.001
**Urban residence, n (%)**	769 (33.5)	860 (37.4)	862 (37.5)	946 (41.2)	916 (39.9)	<0.001
**Smoking, n (%)**	663 (29.0%)	655 (28.6%)	656 (28.6%)	763 (33.3%)	776 (33.8%)	<0.001
**Alcohol drinking, n (%)**	720 (31.6%)	754 (33.1%)	799 (35.2%)	839 (36.9%)	863 (37.9%)	<0.001
**Energy, Kcal/d**	1963.7 ±563.2	2037.8 ±509.6	2125.4 ±508.2	2213.8 ±511.9	2285.2 ±548.4	<0.001
**Fat, Kcal/d**	52.3 ±24.8	62.8 ±23.7	71.3 ±25.2	78.1 ±26.5	87.8 ±31.7	<0.001
**Carbohydrate, Kcal/d**	310.9 ±117.9	300.8 ±101.3	299.4 ±95.7	301.5 ±92.3	296.6 ±95.5	<0.001
**Protein, Kcal/d**	59.8 ±20.0	63.7 ±15.9	67.4 ±16.8	71.0 ±18.2	72.1 ±21.1	<0.001
**Dietary CoQ10, mg/day**	1.6 ±0.6	3.1 ±0.3	4.3 ±0.4	5.9 ±0.6	10.0 ±3.8	<0.001
**Education, n (%)**						
Illiteracy	608 (27.2%)	445 (19.7%)	371 (16.5%)	310 (13.8%)	302 (13.3%)	<0.001
Primary school	423 (19.0%)	449 (19.9%)	436 (19.4%)	408 (18.1%)	442 (19.5%)	
Lower middle school	647 (29.0%)	740 (32.8%)	762 (33.9%)	786 (35.0%)	791 (34.9%)	

Upper middle school and above	554 (24.8%)	623 (27.6%)	680 (30.2%)	744 (33.1%)	729 (32.2%)	
**Occupation, n (%)**						
Unemployed	550 (23.9%)	579 (25.2%)	536 (23.3%)	557 (24.2%)	594 (25.9%)	<0.001
Farmer	1083 (47.1%)	893 (38.9%)	814 (35.4%)	677 (29.5%)	665 (29.0%)	
Worker	302 (13.1%)	412 (17.9%)	488 (21.2%)	516 (22.5%)	457 (19.9%)	
Other	363 (15.8%)	414 (18.0%)	460 (20.0%)	548 (23.8%)	581 (25.3%)	
**Physical activity, n (%)**						
Low	769 (33.5%)	828 (36.0%)	744 (32.4%)	769 (33.5%)	720 (31.3%)	0.006
Moderate	721 (31.4%)	756 (32.9%)	790 (34.4%)	790 (34.4%)	773 (33.7%)	
High	808 (35.2%)	714 (31.1%)	764 (33.2%)	739 (32.2%)	804 (35.0%)	

Supplementary Table S3. Dietary intake of different-sourced CoQ10 and energy-adjusted CoQ10 (mg/d) in study participants.

	Mean	SD	Percentile5	Percentile25	Median	Percentile75	Percentile95
**Total CoQ10 intake**	4.99	3.40	1.14	2.82	4.29	6.36	10.88
**Energy-adjusted total CoQ10 intake**	4.99	3.33	1.22	2.88	4.33	6.32	10.75
**Meats and their processed foods source**	1.86	1.60	0.00	0.64	1.63	2.70	4.75
**Energy-adjusted intake**	1.86	1.57	-0.13	0.73	1.64	2.68	4.61
**Eggs source**	0.06	0.06	0.00	0.01	0.04	0.08	0.16
**Energy-adjusted intake**	0.06	0.06	0.00	0.01	0.04	0.08	0.16
**Dairy products source**	0.02	0.06	0.00	0.00	0.00	0.00	0.11
**Energy-adjusted intake**	0.02	0.06	-0.01	0.00	0.00	0.01	0.11
**Fishes and shellfish source**	0.03	0.23	0.00	0.00	0.00	0.00	0.04
**Energy-adjusted intake**	0.03	0.23	0.00	0.00	0.00	0.00	0.04
**Oils source**	2.58	3.16	0.00	0.57	1.59	3.42	8.39
**Energy-adjusted intake**	2.58	3.15	-0.12	0.59	1.60	3.42	8.29
**Nuts and seeds source**	0.02	0.11	0.00	0.00	0.00	0.00	0.01
**Energy-adjusted intake**	0.02	0.11	0.00	0.00	0.00	0.00	0.01
**Vegetables and their processed foods source**	0.42	0.33	0.04	0.20	0.35	0.55	1.00
**Energy-adjusted intake**	0.42	0.32	0.05	0.21	0.35	0.55	0.99
**Fruits and their processed foods source**	0.02	0.05	0.00	0.00	0.00	0.01	0.11
**Energy-adjusted intake**	0.02	0.05	-0.01	0.00	0.00	0.02	0.11

Supplementary Table S4. The association between diversity score of dietary CoQ10 source and new-onset hypertension.

Diversity score	Model 1		Model 2	
	HR (95%CI)	*p* value	HR (95%CI)	*p* value
As continuous variable				
	0.66 (0.64, 0.68)	<0.001	0.67 (0.65, 0.70)	<0.001
As categorical variable				
Group 1 (<2)	*Ref*		*Ref*	
Group 2 (2)	0.52 (0.48, 0.57)	<0.001	0.55 (0.51, 0.60)	<0.001
Group 3 (3)	0.41 (0.36, 0.46)	<0.001	0.42 (0.37, 0.48)	<0.001
Group 4 (≥4)	0.34 (0.26, 0.43)	<0.001	0.35 (0.26, 0.47)	<0.001
p for trend	<0.001		<0.001	

[a] The CoQ10 intake was adjusted by total energy using the residual method.

[b] Model 1: adjusted for age, sex and BMI.

[c] Model 2: adjusted for age, sex, BMI, job, education level, region, smoking status, alcohol drinking status, baseline SBP, physical activity, abdominal obesity, as well as total energy intake (kcal/day), total dietary CoQ10 intake (mg/day).

Supplementary Table S5. Sensitivity analyses for the association between diversity score of dietary CoQ10 source and new-onset hypertension.

Diversity score	Analysis 1<br>Model 2<br>HR (95% CI)	*p* value	Analysis 2<br>Model 2<br>HR (95% CI)	*p* value	Analysis 3<br>Model 2<br>HR (95% CI)	*p* value
As continuous variable						
	0.65 (0.63, 0.68)	<0.001	0.73 (0.70, 0.76)	<0.001	0.66 (0.63, 0.68)	<0.001
As categorical variable						
Group 1 (<2)	*Ref*		*Ref*		*Ref*	
Group 2 (2)	0.49 (0.45, 0.54)	<0.001	0.60 (0.55, 0.66)	<0.001	0.50 (0.46, 0.54)	<0.001
Group 3 (3)	0.41 (0.36, 0.47)	<0.001	0.54 (0.47, 0.61)	<0.001	0.42 (0.37, 0.47)	<0.001
Group 4 (≥4)	0.28 (0.20, 0.38)	<0.001	0.34 (0.25, 0.46)	<0.001	0.31 (0.24, 0.41)	<0.001

[a] Model 2: adjusted for age, sex, BMI, job, education level, region, smoking status, alcohol drinking status, baseline SBP, physical activity, abdominal obesity, as well as total energy intake (kcal/day), total dietary CoQ10 intake (mg/day).

[b] Analysis 1: The follow-up time of participants with new-onset hypertension were recalculated as the years from the baseline to the first hypertension diagnosis.

[c] Analysis 2: Participants who developed hypertension in the first two years of follow-up were excluded.

[d] Analysis 3: The analyses were conducted among all participants after multiple imputations of missing covariates by chained equation was performed

Supplementary Table S6. The association between diversity score of CoQ10 and new-onset hypertension after the removal of any one kind of dietary CoQ10 sources.

Diversity score	Model 1	*p* value	Model 2	*p* value
	HR (95% CI)		HR (95% CI)	
Excluding meats and their processed foods	0.63 (0.60, 0.65)	<0.001	0.64 (0.62, 0.67)	<0.001
Excluding egg	0.66 (0.64, 0.69)	<0.001	0.67 (0.64, 0.69)	<0.001
Excluding dairy products	0.66 (0.64, 0.68)	<0.001	0.67 (0.65, 0.70)	<0.001
Excluding fishes and shellfish	0.66 (0.63, 0.68)	<0.001	0.67 (0.64, 0.69)	<0.001
Excluding plant-sourced cooking oils	0.65 (0.63, 0.68)	<0.001	0.67 (0.64, 0.69)	<0.001
Excluding nuts and seeds	0.65 (0.63, 0.68)	<0.001	0.66 (0.64, 0.69)	<0.001
Excluding vegetables and their processed foods	0.61 (0.58, 0.63)	<0.001	0.61 (0.59, 0.64)	<0.001
Excluding fruits and their processed foods	0.66 (0.64, 0.69)	<0.001	0.68 (0.65, 0.70)	<0.001

[a] Model 1: adjusted for age, sex and BMI.

[b] Model 2: adjusted for age, sex, BMI, job, education level, region, smoking status, alcohol drinking status, baseline SBP, physical activity, abdominal obesity, as well as total energy intake (kcal/day), total dietary CoQ10 intake (mg/day).
