# Supplementary figures and images for: The Association between the Diversity of Coenzyme Q10 Intake from Dietary Sources and the Risk of New-Onset Hypertension: A Nationwide Cohort Study
Source: Nutrients. 2024 Mar 31;16(7):1017. doi: 10.3390/nu16071017 (PMC11013836; doi:10.3390/nu16071017)

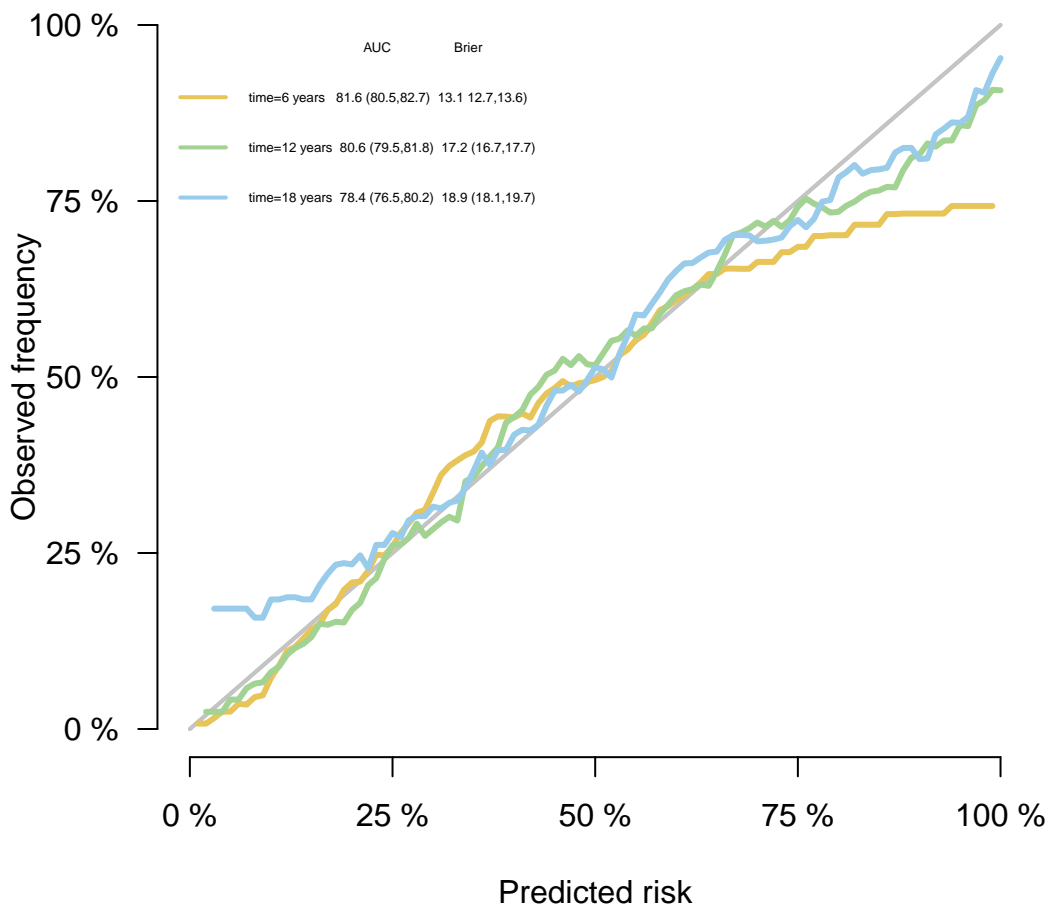

Supplement: Supplementary file 1 [file nutrients-16-01017-s001.zip › Figure S1.pdf]
